# Supplementary material for: Exploring Definitions of “Addiction” in Adolescents and Young Adults and Correlation with Substance Use Behaviors
Source: Int J Environ Res Public Health. 2022 Jun 30;19(13):8075. doi: 10.3390/ijerph19138075 (PMC9266281; doi:10.3390/ijerph19138075)
Supplement: Supplementary file 1 [file ijerph-19-08075-s001.zip › ijerph-1753119-supplementary.pdf]

Table S1. Characteristics of study participants by age group, PACE Vermont Study 2019 (n = 1,110)

	**Ages 12-17** n (%)	**Ages 18-25** n (%)	**Total** n (%)	**p-value**[a]
Overall	372 (34)	738 (66)	1110 (100)	
Major addiction themes				<0.001
Psychological changes	128 (36)	153 (22)	281 (26)	
Physiological Changes	220 (62)	498 (70)	718 (67)	
Behavioral changes	10 (3)	56 (8)	66 (6)	
Sex assigned at birth				<0.001
Male	138 (37)	173 (24)	311 (28)	
Female	233 (63)	564 (77)	797 (72)	
Race/ethnicity, 3 categories				0.399
White	333 (90)	644 (87)	977 (88)	
Non-white/other race	22 (6)	60 (8)	82 (7)	
Hispanic	16 (4)	34 (5)	50 (5)	
Ever substance use				
Any substance[b]	144 (39)	706 (96)	850 (77)	<0.001
Cigarettes	31 (8)	365 (50)	396 (36)	<0.001
Electronic vapor products	81 (22)	461 (63)	542 (49)	<0.001
Alcohol	126 (34)	693 (94)	819 (74)	<0.001
Marijuana	63 (17)	552 (75)	615 (55)	<0.001
Past 30-day substance use				
Any substance[b]	76 (20)	609 (83)	685 (62)	<0.001
Cigarette	11 (3)	126 (17)	137 (12)	<0.001
Electronic vapor products	42 (11)	199 (27)	241 (22)	<0.001
Alcohol	52 (14)	550 (75)	602 (54)	<0.001
Marijuana	35 (10)	281 (38)	316 (29)	<0.001

[a] p-value from chi-square test

[b] Any substance use was defined as use (ever or past 30-day) of any of the following substances: cigarettes, electronic vapor products (EVP), marijuana, or alcohol.
